# The Impact of Parasitic Infestation on Nutritional Status and Micronutrients among Children

**DOI:** 10.1155/2024/6996968

**Published:** 2024-03-28

**Authors:** Usama M. Alkholy, Sherief M. El Gebaly, Walaa E. M. A. Morsi, Waleed E. Elawamy, Samia E. Etewa, Asmaa M. Yousef

**Affiliations:** ^1^Department of Pediatrics, Faculty of Medicine, Zagazig University, Zagazig 44511, Sharqyia, Egypt; ^2^Department of Pediatrics, Faculty of Medicine, Cairo University, Giza, Giza, Egypt; ^3^Department of Medical Parasitology, Faculty of Medicine, Benha University, Benha 13512, Qalioubyia, Egypt; ^4^Department of Medical Parasitology, Faculty of Medicine, Zagazig University, Zagazig 44511, Egypt

## Abstract

**Background:**

Micronutrient deficiency is a great problem that is augmented by infection and poor nutrition. Iron, zinc, and selenium are trace elements needed for human growth.

**Objective:**

To investigate the impact of parasitic infection on nutritional status and serum iron, zinc, and selenium in children attending Pediatrics Outpatient Clinic of Zagazig University Hospitals. *Subjects and Methods*. A case-control study included 140 parasitic infected children and one hundred age- and sex-matched controls. Anthropometric measures were evaluated using specific Egyptian growth charts. Parasites were detected in stool specimens using standard microscopic methods. Atomic absorption spectrophotometer was used for the detection of serum iron, zinc, and selenium. To examine the statistical relationship between intestinal parasitic infection and the relevant variables (gender, residence, socioeconomic status, and age group), the nonparametric chi-square (*χ*^2^) test was used. Data were analyzed statistically using SPSS version 25.

**Results:**

Parasitic infected children showed a statistically significant low weight for age, height for age, and BMI. Serum iron, zinc, and selenium were significantly lower in parasitic infected children than controls. Serum iron, zinc, and selenium have significant positive correlations with weight, height, and BMI, respectively.

**Conclusion:**

Studied serum micronutrients especially zinc and iron and anthropometric indices were significantly lower in parasitically infected children.

## 1. Introduction

Even though they are only required in very small amounts, micronutrients, which include vitamins and minerals, are essential for the regular growth, development, and performance of the human body. These are essential throughout life, but the years of childhood and adolescence are particularly important because of the fast growth and development they characterized [1]. The most common of which are selenium, zinc, and iron [2] because they generally serve as modulators in various chemical, biological, catalytic, and metabolic processes [3]. They are all crucial immune system cofactors that contribute to the control of antioxidant stress. Additionally, iron supports a number of physiological functions, including DNA synthesis, DNA repair, oxygen transport, brain function, and gene regulation [4]. The DNA's structural integrity is specifically maintained and made easier to replicate by zinc [5]. The neurological, musculoskeletal, cardiovascular, endocrine, immune, and reproductive systems all depend on selenium, which is found in the form of selenoproteins and performs a number of metabolic and physiological actions [6].

Nearly one-third of the world's population, or two billion people, suffer from micronutrient deficiencies [7]. They are exacerbated by illnesses such as infection with various parasites eventually resulting in a shortage of valuable trace elements, which are caused by improper consumption of various trace elements, and obstructing its adequate absorption [8].

Each year, intestinal parasite infections impact around 3.5 billion people globally and cause more than 450 million health issues, such as diarrhea, undernutrition, general weakness, and stunted growth and physical development [9].

Environmental factors like nutrition (which is influenced by infection) and disease have an impact on children's growth [10]. Weight and height remain the only anthropometric measurements that are consistently verified and authorized for assessing nutritional status, particularly in children, and predicting performance [11].

Micronutrient deficiency can indeed be common among children, particularly in regions with limited access to diverse and nutritious food sources. When children are infected with parasites, the risk of micronutrient deficiency can be further exacerbated due to various factors such as reduced nutrient absorption, increased nutrient requirements, and nutrient loss through diarrhea or blood loss [12].

The purpose of this study was to examine how the nutritional status and serum levels of iron, zinc, and selenium in preschool and school-aged patients of the Pediatrics Outpatient Clinic at Zagazig University Hospitals were affected by parasite infection.

## 2. Subjects and Methods

### 2.1. Study Area

This research was performed in Pediatrics Outpatient Clinic of Zagazig University Hospitals.

### 2.2. Study Design

A case-control study was undertaken over one year from February 2021 to February 2022.

### 2.3. Subject Sampling

The participants of this work were 240 children: 140 children infected with different parasites known by their positive stool analysis; 89 were males and 51 were females. The 100 children free parasites were considered as a control group; 62 were males and 38 were females with matched age and sex. They were enrolled from children attending Pediatrics Outpatient Clinic of Zagazig University Hospitals. The following inclusion and exclusion criteria were applied.

### 2.4. Inclusion Criteria

Age from 2 up to 18 years, children of both sexes, underweight with apparent undergrowth signs, or normal weight were only considered and those who have no history of vitamins or minerals received for the 6 months preceding the study.

### 2.5. Exclusion Criteria

Age under 2 and older than 18 years; obesity; immunocompromised febrile children at the time of the study; underweight due to chronic conditions like congenital heart problems, endocrine disorders, and tumors; children who had taken dietary supplements containing minerals and vitamins within the previous 6 months; and kids who had received a blood transfusion or an iron-containing medication within the 3 months prior to the study are all excluded.

Both groups were submitted to the following.

### 2.6. Case History

Complete history taking was performed including personal complaints (abdominal pain, diarrhea, flatulence, vomiting, and pruritis ani); perinatal development; and past, present, dietetic, and family history.

### 2.7. Clinical Examination

At the time of diagnosis, data on each patient's demographics and clinical factors were gathered. A thorough clinical examination was performed, with a focus on growth evaluation using anthropometric measurements, detection of anemia-related symptoms including pallor, evidence of micronutrient deficiencies such as dermatitis and hair loss, and the exclusion of any other systemic diseases.

### 2.8. Detection of Parasites in Stools

Stool samples from the same person were taken three times in a row using conventional methods and sterile, leak-proof feces cups. All participant children's details, including name, age, sex, serial number, and day of sample collection, were printed in large print on the stool cups. To determine the stool's color (yellowish, greenish, or brownish), consistency (watery, loose, or well-formed stool), odor (offensive or not), and presence of blood or mucus, the feces was visually inspected. Direct wet smears that were stained with Lugol's iodine were conducted immediately following sample collection. The stool samples were then subjected to the formalin-ethyl acetate sedimentation procedure and evaluated using a direct wet smear and Lugol's iodine-stained smear [13]. The modified Ziehl-Neelsen and trichrome stains were used in staining stool permanent mounts [14]; the parasitological examination was performed at the laboratory of Medical Parasitology Department of Faculty of Medicine, Zagazig University. According to their parasitological stool results, parasitically infected children were differentiated to protozoa, helminths, and mixed infected cases, but parasitically free cases were considered controls.

The cellophane-tape perianal swab method was used to find eggs of *Enterobius vermicularis* [15], which was performed by parents in the morning, according to our instructions. The Medical Parasitology Department, Faculty of Medicine, Zagazig University, received the collected samples, which were subsequently brought there for light microscopy analysis. On three consecutive days, the same detection process was used. If eggs were discovered, *E. vermicularis* infection in children was considered to have occurred.

### 2.9. Nutritional Status Assessment

The children stood on the ground without shoes, their backs on the vertical backboard, and their buttocks and shoulders touching it. The same person took the mean of three separate measurements of height and weight. The following anthropometric indices were used to gauge the kids' nutritional status: for the children in the study, the body mass index (BMI) was computed by dividing the child's weight in kilograms by the square of height in meters (kg/m^2^). All measures were placed on an Egyptian growth chart according to age and gender [16].

### 2.10. Biochemical Estimation

Blood samples were taken in a sterile tube to estimate the amounts of iron, zinc, and selenium in the serum. The central laboratory of the Zagazig University Hospital centrifuged three milliliters of blood for 15 minutes. One cc was taken from each resulting serum sample and kept in Eppendorf tubes at 80°C until the assay. In the central laboratory of the Faculty of Veterinary at Zagazig University, we used a Buck Scientific 210 VGP Atomic Absorption Spectrophotometer to measure the levels of iron, zinc, and selenium in serum samples. The instrument was calibrated using the following units of measurement: g/dL for iron, g/mL for zinc, and g/L for selenium [17].

### 2.11. Statistical Analysis

Using IBM SPSS Statistics for Windows version 25, the collected data were tabulated and analyzed. Descriptive statistics were used to report the prevalence, which is defined as the percentage of positive results compared to all samples that were examined [18]. To examine the statistical relationship between intestinal parasitic infection and the relevant variables (gender, residence, socioeconomic status, and age group), the nonparametric chi-square (*χ*^2^) test was used. Student's *t*-test and ANOVA test were also used. *P* value was set at >0.05 for nonsignificant results, <0.05 for significant results, and <0.001 for highly significant results.

Sample size was calculated using OpenEpi according to the following:

The total number of children coming to Zagazig University Hospital in 6 months is expected to be 645 cases, and the prevalence of parasitic infection was 55% [19]. At a 95% confidence interval, the calculated sample size is 240.

## 3. Results

Demographic criteria of the studied groups showed that the mean age of infected cases was 13.2 ± 5.5 years while the mean age of noninfected cases was 12 ± 4.1 years with insignificant statistical difference between the two groups. Males represented the majority of the infected and noninfected groups (63.6% and 62%), respectively, with insignificant statistical differences. The majority of the infected and noninfected group inhabited the rural areas (66.4% and 56%), respectively, with insignificant statistical differences (Table 1).

According to Table 2, parasitic infections were detected in infected children as follows: protozoal infections were detected in 32.1% (45/140 cases). The protozoal parasites recovered from stool examination of the studied groups were *Giardia lamblia* with a total prevalence of 8.6% (12 cases) as the most common parasite. The second common protozoan infecting children was *Entamoeba histolytica/dispar* with a prevalence of 7.1% (10 cases). The third common protozoan was *Cryptosporidium* spp. with a prevalence of 6.4% (9 cases), *Entamoeba coli* infected 7 cases (5%), and the prevalence of *Blastocystis hominis* in our study is 3.6%. *Cyclospora cayetanensis*, an opportunistic spore-forming parasite, was detected in 1.4% of children.

Helminthic parasitic infections were detected in 48.6% (68/140 cases). The helminthic parasites recovered from stool examination of the studied groups were as follows: *E. vermicularis* 35.7% (50/140 cases), *Hymenolepis nana* 7.9% (11/140), and *Ascaris lumbricoides* 5% (7/140).

**Figure 1:**
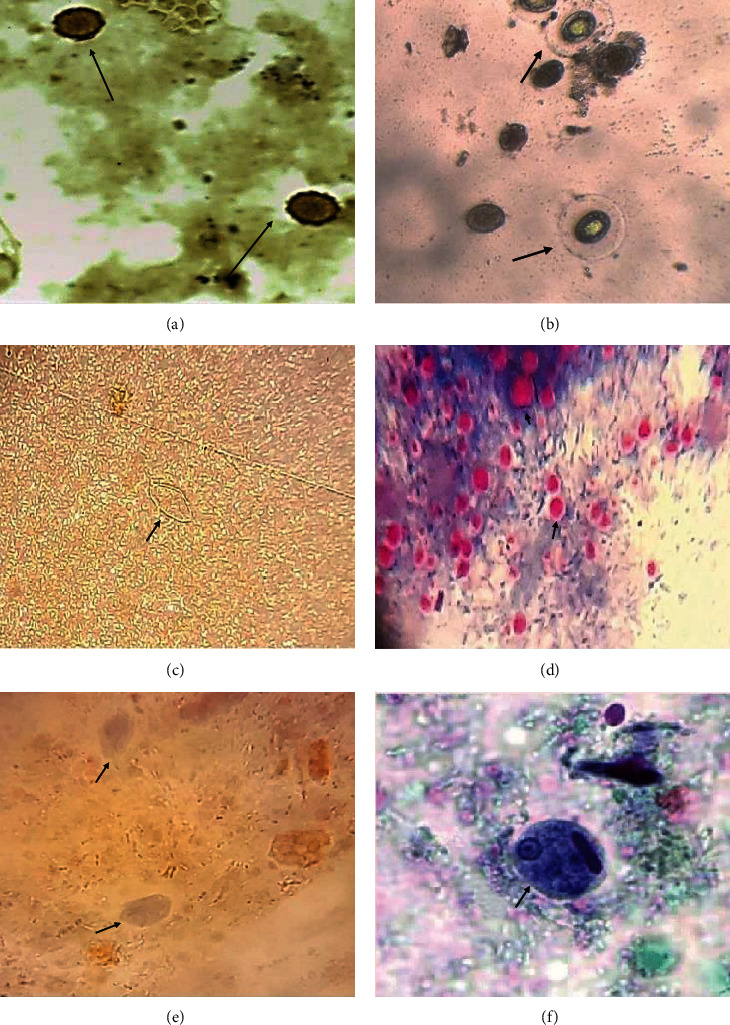
Parasitic infections of the infected children: (a) *Ascaris lumbricoides* egg, iodine stained (×400); (b) *H. nana* egg, iodine-stained smear (×400); (c) *E. vermicularis* egg, wet mount (×400); (d) *Cryptosporidium* oocyst, modified Ziehl-Neelsen stain (×1000); (e) *Giardia lamblia* cyst, trichrome stain (×1000); (f) *Entamoeba histolytica* cyst, trichrome stain (×1000).

Mixed parasitic infections were detected in 19.3% (27/140 cases) being as follows: four cases of *G. lamblia* and *Cryptosporidium* spp. and 7 cases of *G. lamblia* and *E. histolytica/dispar*, while 9 cases of *E. histolytica/dispar* and *E. vermicularis* and 2 cases of *E. histolytica/dispar* and *E. coli*. Also, *G. lamblia* and *H. nana* were detected in 3 cases. *Cryptosporidium* spp. and *A. lumbricoides* were detected in 2 cases (Figure 1).

As shown in Table 3, the majority of the clinical manifestations at the time of examination revealed that the majority of infected children were suffering from abdominal pain, diarrhea, flatulence, vomiting, pruritus ani, pallor, dermatitis, and hair loss (90%, 81.4%, 85%, 75%, 42.14%, 87.1%, 79.3%, and 75%, respectively) with highly significant statistical difference.

Anthropometric measures of the infected and noninfected groups showed that the mean weights of the studied groups were 29.7 ± 5.1 kg and 41.5 ± 8.4 kg, respectively, and the mean heights were 139 ± 6.8 cm and 145 ± 13.2 cm, respectively, with significant statistical differences between the two groups. The mean BMI of the studied groups was 15.07 ± 3.46 kg/m^2^ and 15.93 ± 3.04 kg/m^2^, respectively, with significant statistical differences (Table 4).

The current study showed that there was a statistically significant increase in the frequency of low iron, zinc, and selenium level among the infected case groups compared to the noninfected group as shown in Table 5.

Our study showed that there was a statistically significant decrease in iron, zinc, and selenium level among infected cases compared to the noninfected groups as shown in Table 6).

Table 7 shows that there was a statistically significant decrease in weight, height, and BMI among infected cases compared to the noninfected groups.

Correlations among serum micronutrients and anthropometric measurements in children with parasitic infection are shown in Table 8. Serum iron, zinc, and selenium have significantly positive correlations with weight for age, weight for height, and BMI.

After applying linear regression for significant predictors of micronutrient level among infected children as shown in Table 9, it was found that iron level was significantly correlated with weight, height, and BMI, while both zinc and selenium were significantly correlated with weight and BMI.

## 4. Discussion

Due to their morbid nature and the considerable health risks they represent, parasitic infection is the primary cause of sickness and illness throughout the world [20]. Micronutrient deficiencies are one of several health issues in underdeveloped nations that are exacerbated by infectious diseases and inadequate nutrition, producing a complex cycle in children due to their high demands and rapid development rate [21]. Inappropriate consumption of micronutrients, along with obstacles to adequate absorption, which were exacerbated by parasite infection, ultimately resulted in a lack of important trace elements [8].

So, this cross-sectional study was aimed at analyzing how children's anthropometric measurements and micronutrient levels are affected by intestinal parasitic infection.

Our study was conducted on 240 cases aged between 2 and 18 years of both genders and from both rural and urban areas.

In our study, we found that sociodemographic characteristics of the studied groups revealed no significant statistical differences in the infected and noninfected groups.

The results of our study recorded that the mean age among infected children was 13.2 ± 5.5 while among the noninfected groups was 12 ± 4.1 with insignificant statistical difference which is in accordance with a report that found high frequency of intestinal parasitic infection among those aged 11 to 20 (65.6%) [22]. Another report supported this finding and suggested that the increased risk of infection in this age group was explained by their close interaction with the polluted environment [23]. According to a series of studies, children under 10 are more prone to parasite infection, which may be related to their unhygienic behaviors [24].

Regarding the gender, our findings revealed that males were more susceptible to parasitic infection (63.6%) than females (36.4%) with insignificant statistical difference. In consensus, it was reported that there were greater infection rates in male cases (66.6%) compared to female cases (41.4%) [22]. In internal reports [25] as in Tanta city in Egypt and in external reports [20] as in Debre Berhan town in Ethiopia, male children had greater rates of intestinal parasitic infections. On the other hand, some studies claim that female individuals had a higher rate of intestinal parasite infection than male participants [26]. Nevertheless, other report [27] refused the concept that age or genders are related to intestinal parasitism.

As regards residence, we found that the majority of infected children were living in rural areas (66.4%) than urban areas (33.6%) with nonsignificant statistical difference. Accordingly, the prevalence of parasitic infection among rural children was higher than that of urban children (66.1%, 33.9%), respectively [25], and the widespread use of water from the Nile for drinking, bathing, and recreation and the difficulty of changing these sociocultural practices were cited in this report as the causes of the higher incidence of intestinal parasites in rural populations in Egypt.

Among the intestinal protozoa found, the most frequently detected species were *G. lamblia* (8.6%), *E. histolytica/dispar* (7.1%), *Cryptosporidium* spp. (6.4), *E. coli* (5%), and *B. hominis* (3.6%). 1.4% of children had *C. cayetanensis*, an opportunistic spore-forming parasite. Earlier reports that were done in Egypt found that *G. lamblia* (26%) and *E. histolytica* (13.4%) were found to be the most common parasite diseases among school children in the Zagazig district of northeastern Egypt [28]. In the Greater Cairo region of Northern Egypt, *G. lamblia* was the most prevalent parasite infection (26%) [29]. The possibility that Egyptians are exposed to the same risk factors, such as eating soiled vegetables, having inadequate water treatment, and having poor hygiene practices, could explain the similarities between the parasitic species discovered during the current study and those discovered during previous related studies conducted in Egypt.

Various studies conducted outside of Egypt also supported our findings. A study in a tertiary care hospital in Nepal reports that the most common parasitic infections were *G. lamblia* (3.34%), followed by *E. histolytica/dispar* (1.96%) [30]. Similarly, In Ethiopia, the most common parasites found among asymptomatic food handlers were *E. histolytica* (5.5%) followed by *G. lamblia* (3%) [31].

*E. vermicularis* was the most often recovered helminth (35.7%), followed by *H. nana* (7.9%), among the parasitic species identified during our research. An Egyptian study conducted in Tanta found that *E. vermicularis* and *H. nana* were the two most common helminthic infections among the primary school children [25].

In contrast to the research that showed that *A. lumbricoides* was one of the major parasitic infections among school children in Zagazig region, Northeastern Egypt, *A. lumbricoides* was one of the fewest parasites recovered in the current study (5%) [22].

The prevalence of mixed infections was 19.3% in our study; all of them had double infections as mentioned before. Similar studies observed mixed infection prevalence rates in the form of *G. lamblia*+*E. coli* (2%), *G. lamblia*+*E. vermicularis* (1.75%), *E. histolytica/dispar*+*E. vermicularis* (1%), *G. lamblia*+*E. histolytica/dispar* (0.5%), and *G. lamblia*+*E. vermicularis*+*H. nana* (0.25%) [32]. Poor cleanliness and the fact that numerous parasite species share the same route of transmission could be responsible for the multiple or mixed infections that have been detected [33].

Regarding anthropometric measurements, it was discovered that there was a substantial difference between the infected and noninfected groups in terms of weight, height, and BMI. According to a different study, the infected group had statistically significant lower weight-for-height, weight-for-age, and body mass index (BMI) values than the noninfected group [2]. Mohammed Mealy et al. [25], in a comparable way, found a significant difference in BMI between healthy and infected subjects. This finding can be explained by the intestinal tissue damage driven by parasite infection as well as other nutrient deficiencies that could affect weight gain and other children's health issues [34]. Our findings contradict another Nigerian study revealing no statistically significant relationship between parasite infection and nutritional status [35].

Our findings were consistent with the study's findings regarding the association between intestinal parasites and clinical symptoms at the time of examination, which indicated that the majority of infected children experienced abdominal pain, dysentery, vomiting, perianal itching, diarrhea, and pallor (69.9%, 9.1%, 3.2%, 33.9%, 12.9%, and 48.4%, respectively), with a highly significant statistical difference [25]. Most of these symptoms were general signs of abdominal discomfort like nausea, vomiting, diarrhea, loss of appetite, and weight loss. Itchy anus in *E. vermicularis* and *Dipylidium caninum* infections, or jaundice accompanied by fever and swollen liver in instances of *Fasciola hepatica*, were clinical symptoms peculiar to the parasite that were identified in a small number of cases [36]. Children without symptoms (26.7%, 267 out of 996) exhibited a significantly higher prevalence (*P* < 0.001) of intestinal parasites compared to children with symptoms [37]. This finding suggests the possibility of a high carrier state rate or a low parasite burden that often goes undetected and untreated, potentially contributing to the observed difference [38].

Children who were infected in the current study had considerably lower serum levels of zinc, iron, and selenium than controls. Another study found that the serum levels of trace elements such as magnesium, zinc, and selenium were reduced by enterobiasis and G. lamblia infection [39]. Numerous studies that elucidated how intestinal parasites promote poor absorption of a number of minerals also corroborated these findings [40].

According to certain research, people with parasitic infections not only lacked certain micronutrients, but their serum levels also rose after taking antiparasitic drugs [41]. Another report observed that three months following treatment of patients infected by *E. vermicularis* and *G. lamblia* infections, serum selenium, zinc, and magnesium deficits had dramatically improved. Patients with G. lamblia infections reportedly had considerably lower serum zinc levels than kids who were parasite-free [42–44]. *G. lamblia* variant-specific surface proteins bind zinc and other heavy metals in the colon, producing zinc malabsorption during giardiasis [45]. Nevertheless, some studies have suggested that this decrease may be brought on by impairments and mucosal damage, which, taken alone, could limit the absorption of zinc [41, 46], and consequently, this interference may affect the activity of digestive enzymes like lipases, proteases, and disaccharidases as well as the release of cytopathic substances that harm the intestinal epithelium [47]. It is hypothesized that the enhanced intestinal absorption of zinc linked with antigiardiasis drugs may be due to the repair of the intestinal mucosa that had been damaged by *G. lamblia* [48]. According to Çulha and Sangün [49], there was no appreciable difference in zinc levels between giardiasis patients and controls.

The results are consistent with other research that discovered that children with parasite infection had low iron levels in their sera, as blood iron levels were significantly lower in children with parasitic infection than in children in the control group in the current study [41, 50]. The iron deficiency in this study could be due to a number of factors including a combination of decreased consumption and insufficient absorption. Because iron is essential for parasite growth and reproduction, its usage by parasites cannot be disregarded [51].

As regards the serum selenium level, it was noticeably lower in parasitic infected children than it was in the control group. Another study found compelling evidence linking helminthiasis to lower serum concentrations of selenium and other numerous crucial trace elements [52].

The current study's findings showed strong positive correlations among weight, height, and BMI along with levels of iron, zinc, and selenium. According to other studies, there are strong positive relationships between serum micronutrients and anthropometric variables (weight, height, and BMI) [2, 21], and this is supported by research showing a link between blood selenium levels and motor function in 9.6-year-old Bangladeshi children [53].

As evidenced by a significant decrease in the *Z*-score for weight, height, and head circumference in the *Giardia*-infected children, it has been observed that a lack of trace elements has an effect on children's growth [54]. Another study found that serum zinc levels significantly influenced anthropometric measurements, especially body weight and BMI [55].

According to research on the effects of zinc shortage on anthropometric measurements, zinc deficiency has an impact on circulating IGF-1 levels regardless of total caloric intake [56]. It is possible that low zinc levels correspond to lower IGF-1 levels. Perhaps a deficiency in zinc reduces the liver's ability to express the growth hormone (GH) receptor and GH binding protein, which lowers IGF-1 levels [57].

## 5. Conclusion

In comparison to the control group, children with parasite infection had significantly decreased serum levels of the micronutrients iron, zinc, and selenium. The anthropometric parameters and the serum levels of iron, zinc, and selenium were found to be positively correlated. It is advised that further research be done to clarify the connections between parasite illness, micronutrients, cognition, and child development.

## Figures and Tables

**Table 1:** Demographic criteria of the infected and noninfected groups.

Variable	Infected (*N* = 140)	Noninfected (*N* = 100)	Test	*P* value
*Gender*				
Male	89 (63.6%)	62 (62%)	*χ* ^2^ (0.06)	0.80 NS
Female	51 (36.4%)	38 (38%)		
*Residence*				
Rural	93 (66.4%)	56 (56%)	*χ* ^2^ (2.70)	0.10 NS
Urban	47 (33.6%)	44 (44%)		
*Age (years)*				
Range	2-18	2-18	*t*-test (1.85)	0.07 NS
Mean ± SD	13.2 ± 5.5	12 ± 4.1		

SD: standard deviation; *t*: independent *t*-test; *χ*^2^: chi-square test; NS: nonsignificant (*P* > 0.05).

**Table 2:** Prevalence of different parasitic infections among the infected groups (*N* = 140).

Parasite species	Number (no.)	Percentage (%)
I. Protozoa	45	32.1%
*Giardia lamblia*	12	8.6%
*Entamoeba histolytica/dispar*	10	7.1%
*Cryptosporidium* spp.	9	6.4%
*Entamoeba coli*	7	5%
*B. hominis*	5	3.6%
*Cyclospora*	2	1.4%
II. Helminths	68	48.6%
*Enterobius vermicularis*	50	35.7%
*Hymenolepis nana*	11	7.9%
*Ascaris lumbricoides*	7	5%
III. Mixed infection	27	19.3%
*Giardia lamblia* and *Cryptosporidium* spp.	4	2.9%
*Giardia lamblia* and *Entamoeba histolytica*	7	5%
*Entamoeba histolytica* and *Enterobius vermicularis*	9	6.4%
*E. histolytica+Entamoeba coli*	2	1.4%
*Giardia lamblia* and *Hymenolepis nana*	3	2.1%
*Cryptosporidium* spp. and *Ascaris lumbricoides*	2	1.4%
Total	140	100%

**Table 3:** Distribution of clinical manifestations among the infected and noninfected groups.

	Infected (*N* = 140)	Noninfected (*N* = 100)	*χ* ^2^	*P*
	No. (%)			
Abdominal pain (*N* = 145)				
+ve	126 (90%)	19 (19%)	123	<0.001^∗∗^
-ve	14 (10%)	81 (81%)		
Diarrhea (*N* = 126)				
+ve	114 (81.4%)	12 (12%)	112.8	<0.001^∗∗^
-ve	26 (18.6%)	88 (88%)		
Flatulence (*N* = 142)				
+ve	119 (85%)	23 (23%)	92.81	<0.001^∗∗^
-ve	21 (15%)	77 (77%)		
Vomiting (*N* = 121)				
+ve	105 (75%)	16 (16%)	81.23	<0.001^∗∗^
-ve	35 (25%)	84 (84%)		
Pruritis ani (*N* = 59)				
+ve	59 (42.14%)	0 (0%)	55.88	<0.001^∗∗^
-ve	81 (57.85%)	100 (100%)		
Pallor (*N* = 136)				
+ve	122 (87.1%)	14 (14%)	127.1	<0.001^∗∗^
-ve	18 (12.9%)	86 (86%)		
Dermatitis (*N* = 122)				
+ve	111 (79.3%)	11 (11%)	108.8	<0.001^∗∗^
-ve	29 (20.7%)	89 (89%)		
Hair loss (*N* = 112)				
+ve	105 (75%)	7 (7%)	108.4	<0.001^∗∗^
-ve	35 (25%)	93 (93%)		

*χ* ^2^: chi-square test. ^∗∗^Highly significant (*P* < 0.001).

**Table 4:** Anthropometric measures of the parasitic infected and noninfected groups.

Variable	Weight (kg)	Height (cm)	BMI (kg/m^2^)	*Z*-score (percentile)
	Mean ± SD			
Infected (*n* = 140)	29.7 ± 5.1	139 ± 6.8	15.07 ± 3.46	-1.1 (7)
Noninfected (*n* = 100)	41.5 ± 8.4	145 ± 13.2	15.93 ± 3.04	-0.2 (44.6)
*t*-test	13.5	4.16	2.04	
*P* value	<0.001^∗∗^	<0.001^∗∗^	0.04^∗^	

SD: standard deviation; *t*: independent *t*-test. ^∗^Significant (*P* < 0.05). ^∗∗^Highly significant (*P* < 0.001).

**Table 5:** Frequency of serum micronutrient deficiency in infected and noninfected children.

Variable	Infected (*N* = 140)	Noninfected (*N* = 100)	*χ* ^2^	*P*
	No. (%)			
Iron level (*μ*g/dL)				
Normal (*N* = 104)	18 (12.9%)	86 (86%)	127.1	<0.001^∗∗^
Low (*N* = 136)	122 (87.1%)	14 (14%)		
Zinc level (*μ*g/mL)				
Normal (*N* = 118)	29 (20.7%)	89 (89%)	108.8	<0.001^∗∗^
Low (*N* = 122)	111 (79.3%)	11 (11%)		
Selenium level (*μ*g/L)				
Normal (*N* = 128)	35 (25%)	93 (93%)	108.4	<0.001^∗∗^
Low (*N* = 112)	105 (75%)	7 (7%)		

*χ* ^2^: chi-square test. ^∗∗^Highly significant (*P* < 0.001).

**Table 6:** Serum micronutrient level in normal and low cases among infected and noninfected children.

Variable	Infected (*N* = 140)	Noninfected (*N* = 100)
	Mean ± SD	
Iron level (*μ*g/dL)		
Normal	107 ± 11.5	126 ± 23.5
Low	43 ± 9.5	61 ± 8.5
*t*-test	25.95	10.2
*P*	<0.001^∗∗^	<0.001^∗∗^
Zinc level (*μ*g/mL)		
Normal	0.82 ± 0.18	1.09 ± 0.13
Low	0.29 ± 0.17	0.51 ± 0.14
*t*-test	14.77	13.85
*P*	<0.001^∗∗^	<0.001^∗∗^
Selenium level (*μ*g/L)		
Normal	79.5 ± 7.6	91.2 ± 2.1
Low	39.8 ± 5.9	61.1 ± 5.2
*t*-test	31.98	31.9
*P*	<0.001^∗∗^	<0.001^∗∗^

SD: standard deviation; *t*: independent *t*-test. ^∗^Significant (*P* < 0.05). ^∗∗^Highly significant (*P* < 0.001).

**Table 7:** Relation between serum micronutrients and anthropometric measures among infected children (*N* = 140).

Variable	*N*	Weight (kg)	Height (cm)	BMI (kg/m^2^)	*Z*-score (percentile)
		Mean ± SD			
Iron level					
Normal	18	44.8 ± 5.8	153 ± 7.3	19.14 ± 4.6	-0.2 (45.3)
Low	122	28.1 ± 6.5	137 ± 5.2	14.97 ± 3.65	-1.6 (8.6)
*t*-test		10.31	11.52	4.37	
*P* value		<0.001^∗∗^	<0.001^∗∗^	<0.001^∗∗^	
Zinc level					
Normal	29	40.5 ± 7.5	149 ± 9.6	18.24 ± 5.6	-0.13 (43)
Low	111	30.1 ± 3.2	136 ± 4.9	16.27 ± 3.1	-1.3 (6.9)
*t*-test		11.27	10.13	2.52	
*P* value		<0.001^∗∗^	<0.001^∗∗^	0.01^∗^	
Selenium level					
Normal	35	49.1 ± 6.8	158 ± 5.4	19.67 ± 4.2	-0.22 (46.1)
Low	105	29.7 ± 4.1	138 ± 6.5	15.60 ± 3.9	-1.5 (7.2)
*t*-test		20.26	16.40	5.24	
*P* value		<0.001^∗∗^	<0.001^∗∗^	<0.001^∗∗^	

SD: standard deviation; *t*: independent *t*-test. ^∗^Significant (*P* < 0.05). ^∗∗^Highly significant (*P* < 0.001).

**Table 8:** Correlations among serum micronutrients and anthropometric measures among infected children (*N* = 140).

Variable	Weight (kg)		Height (cm)		BMI (kg/m^2^)	
	*r*	*P*	*r*	*P*	*r*	*P*
Iron level (*μ*g/dL)	0.58	<0.001^∗∗^	0.34	0.01^∗^	0.41	0.006^∗^
Zinc level (*μ*g/mL)	0.44	0.003^∗^	0.31	0.03^∗^	0.40	0.006^∗^
Selenium level (*μ*g/L)	0.61	<0.001^∗∗^	0.38	0.009^∗^	0.50	<0.001^∗∗^

*r*: Pearson's correlation coefficient. ^∗^Significant (*P* < 0.05). ^∗∗^Highly significant (*P* < 0.001).

**Table 9:** Linear regression analysis for significant predictors of serum micronutrients among infected children (*N* = 140).

Variable	Iron (*μ*g/dL)			Zinc (*μ*g/mL)			Selenium level (*μ*g/L)		
	*B*	SE	*P*	*B*	SE	*P*	*B*	SE	*P*
Weight (kg)	0.60	0.02	<0.001^∗∗^	0.29	0.03	0.04^∗^	0.32	0.01	0.03^∗^
Height (cm)	0.51	0.03	0.006^∗^	0.04	0.18	0.82 NS	0.004	0.002	0.12 NS
BMI (kg/m^2^)	0.42	0.01	0.01^∗^	0.48	0.09	0.003^∗^	0.51	0.02	0.007^∗^

*B*: regression coefficient; SE: standard error; NS: nonsignificant (*P* > 0.05). ^∗^Significant (*P* < 0.05). ^∗∗^Highly significant (*P* < 0.001).

## Data Availability

The biometric data used to support the findings of this study are available from the corresponding author upon request.
